# Assessment of Risk of Possible Exposure to Rabies among Processors and Consumers of Dog Meat in Zaria and Kafanchan, Kaduna State, Nigeria

**DOI:** 10.5539/gjhs.v6n1p142

**Published:** 2013-11-03

**Authors:** Leslie E. Odeh, Jarlath U. Umoh, Asabe A. Dzikwi

**Affiliations:** 1 Department of Veterinary Public Health and Preventive Medicine, Faculty of Veterinary Medicine, Ahmadu Bello University, Zaria, Nigeria

**Keywords:** knowledge, attitudes, practices, processors, consumers, rabies, slaughtered dogs, Nigeria

## Abstract

Canine rabies is endemic in Nigeria. Some of the dogs slaughtered for human consumption may be infected with rabies virus, thus exposing handlers of raw dog meat to the disease since the virus may be present in the nerves in the meat. A cross-sectional study was designed and a structured questionnaire was designed and administered to a convenience sample of 160 processors and consumers (100 from Zaria and 60 from Kafanchan), by face to face interview at the slaughter sites or dog meat sale points. The questionnaire sought information on demographic characteristics of the respondents, rabies knowledge, attitude and actions the respondents would take if exposure occurs. Associations between demographic variables and categorized knowledge, attitude or practice scores were assessed using χ^2^ analysis. The relationship between non-categorized scores was assessed using multiple regression analysis. Also, 154 brain samples from slaughtered dogs (74 from Zaria and 80 from Kafanchan) were checked for rabies antigen using direct fluorescent antibody test. Of the 160 respondents, 49 (30.6%) were involved in the slaughtering and sale of dog meat while 111(69.4%) were involved in handling and consumption of processed dog meat. Only 123(76.9%) knew that dogs are common source of rabies in Nigeria and 105(65.6%) knew that rabies affect humans. Also 110(68.8%) did not have adequate knowledge of the clinical signs of rabies. The level of knowledge, having positive attitudes and knowing acceptable practices were directly proportional to the level of education. Respondents from Kafanchan had higher level of knowledge and more positive attitudes towards rabies than those from Zaria. There were significant correlations between knowledge and attitude scores (r=0.49) and between knowledge and practice scores (r=0.43) at p<0.001. Rabies antigen was detected in the brain of 6 (3.9%) of the slaughtered dogs. The findings indicate that processors and consumers of dog meat are deficient in the knowledge of rabies. There is therefore a need for educational programmes targeted at this high risk group to increase their level of knowledge and reduce the risk of exposure.

## 1. Introduction

Rabies is a fatal encephalitis with almost 100% case fatality rate. In rabid animals, the rabies virus confines mainly to the nerves and adipose tissues eventhough it can also be found in all parts of the body (Srinivasan et al., 2005). Transmission is possible following consumption of carcasses of animals that died of rabies and also by consumption of raw dog meat (Wallersein, 1999). No intervention is effective in stopping the disease after the onset of symptoms. Hence, there is need to prevent the disease. Rabies is transmitted by contamination of wound with infectious material especially saliva from rabid animals and from bites by rabid animals. In Nigeria, the dog is responsible for over 96% of rabies cases in animals (WHO, 2005). Rabies remains an endemic and neglected tropical disease in Nigeria and is often misdiagnosed, under-diagnosed and underreported (Adedeji et al., 2010; Ehizibolo et al., 2011). In some cases, despite proper vaccination, the disease has been reported (Adedeji et al., 2010; Kujul et al., 2010). Some of the factors responsible for the endemicity of rabies in Nigeria include vaccine and vaccination factors, lack of knowledge and information about the disease and poor public awareness, socio-economic factors and increasing human activities involving dogs eg hunting. Dog trade is also gaining popularity in Nigeria. Dogs are collected from villages in northern Nigeria and taken to dog markets especially in Plateau state and from there they are taken to virtually all major towns in Nigeria for slaughter for human consumption (Personal contact with dog dealers). Dog meat is eaten by various ethnic groups in Nigeria. Dog slaughter points are particularly common in Plateau, Cross river, Akwa Ibom, Taraba and Gombe (Simoons, 1994), Kaduna, Kebbi and Ondo (Okonko et al., 2010). Neutralizing antibodies associated with lyssaviruses have been detected in some areas from unvaccinated dogs in Nigeria suggesting dogs in Nigeria may maintain not only rabies but also other lyssaviruses (Ogunkoya et al., 1990; Dzikwi et al., 2010). Various studies have reported the presence of rabies antigen in the brain of some of the dogs slaughtered for human consumption (Ajayi et al., 2006; Akombo, 2009; Aliyu et al., 2010). Thus there exists a risk that rabies could be transmitted to human during slaughtering and processing of dogs for food especially as processors occasionally sustain minor cuts during the process. The issue is whether those involved in dog meat related activities are aware of the risks to which they are exposed. If they are aware, it is expected that their attitudes and practices would change to lower the risk. The cuts or wounds sustained by processors during processing are only rarely reported to medical authorities probably due to inadequate knowledge of the risks involved. There is a need for detailed epidemiological studies and accurate data to be collected following surveillance in order to control this disease in Nigeria (Adedeji et al., 2010).

This study is to assess the risk of exposure to rabies by assessing the rabies related knowledge, attitudes and practices of those involved in dog meat related activities in Zaria and Kafanchan, Kaduna state and checking for evidence of rabies infection in the slaughtered dogs.

## 2. Materials and Methods

### 2.1 Study Design

This cross sectional study was carried out April to September, 2012 in two towns, Zaria and Kafanchan in Kaduna State, Nigeria.

### 2.2 Study Area and Population

Zaria is in the northern part of Kaduna state and located between latitude 11°4'N and longitude 7°42'E (Anon, 2005) has a population of 547,000 inhabitants and a 3.5% annual growth rate (Ministry of Economic Development, 1996). Zaria has many educational institutions, some private veterinary clinics and a university veterinary teaching hospital belonging to the Ahmadu Bello University. It also has two military establishments located at Basawa and Sabon Gari area. There are commercial areas attached to these military establishments where slaughtering of dogs is carried out. Kafanchan is in the southern part of Kaduna state and lies between 9°34'N and 8°18'E and has an estimated population of 83,092 (The World Gazette, 2011). This is a high dog-meat consuming area and many dog slaughtering points exist both in the main town and the surrounding areas. Most of the dogs slaughtered in Kafanchan are brought from dog markets in Plateau state. The respondents were a convenience sample of persons who were seen at dog meat processing and selling points. Only those who were willing to participate in the study were interviewed. Also, samples of the brains of dogs slaughtered on the day of the visit were obtained to check for rabies virus antigen.

### 2.3 Survey Methods

A structured questionnaire was developed and pre- tested on 20 respondents. After validation, the questionnaires were administered to 160 persons who were involved in dog meat processing or consumption using face to face interviews. The interviews were carried out in the dog meat processing or eating points. One hundred (100) respondents were recruited from Zaria and 60 from Kafanchan. The operators and owners of the slaughter sites and sale points were informed of the study and their cooperation sought before sampling was done. All respondents were verbally informed of the purpose of study. Oral consent was obtained. Those who did not give their consent were excluded from the study. The interviews were done in Hausa and English language. The questionnaire had four sections. Section A had 15 items covering demographic characteristics of the population (name, age, sex, occupation, number of children owned, level of education, etc). Section B had 18 questions to assess knowledge about rabies transmission, clinical signs/symptoms, prevention and control. Section C had 9 items on attitudes towards rabies and Section D had 12 questions on practices towards rabies prevention and control.

The questions on knowledge, attitudes and practices required “yes”, “no” and “don’t know/undecided” responses. A marking scheme containing expected correct answers was prepared and used to mark and score the responses. Don’t know/undecided responses were considered as wrong answers. For each correct and incorrect answer one and zero points were assigned respectively. A higher score indicated greater level of knowledge, positive attitudes or acceptable practices.

### 2.4 Detection of Rabies Antigen

One hundred and fifty four (154) heads of dogs (75 from Zaria and 80 from Kafanchan) were purchased from dog meat processors at slaughter points. The brain was removed from each dog head as described by Atanasiu (1975). The samples were tested for rabies antigen by direct fluorescent antibody technique (Dean et al., 1996) in the Department of Veterinary Public Health and Preventive Medicine, Ahmadu Bello University, Zaria, Nigeria.

Briefly impression smears of the hippocampus were fixed in acetone at -20°C, stained with antinucleocapsid monoclonal antibody labelled with fluorescein isothiocynate (Fujirebio Diagnostics Inc. Malvern PA19355, USA) and examined for apple green fluorescence under a fluorescent microscope (MEIJI TECHNO, Japan).

### 2.5 Statistical Analysis

Data obtained was entered into a computer and analyzed using Statistical Package for Social Sciences, SPSS (Version 17, SPSS Inc. Chicago IL USA). Demographic variables were presented using descriptive statistics. Knowledge scores were categorized into inadequate (0-9) and adequate (10-18); attitude scores were categorized into poor (0-4) and good (5-9) and practice scores were categorized as unacceptable (0-5) and acceptable (6-12). Associations between demographic variables and the categorized scores were assessed using χ^2^ test of association and odds ratio; confidence intervals (95%) were calculated for odds ratios. Values of p<0.05 were considered significant in the χ^2^ analysis. Relationships between non-categorized scores were assessed using multiple regression analysis. Brain samples positive for rabies antigen were expressed as a percentage of the total sample examined.

## 3. Results

### 3.1 Demographic Characteristics of the Respondents

Of the 160 respondents, 145(90.6%) were males, 65(40.6%) were between the ages 20 to 30 years, 80(50.0%) were married, only 4(2.5%) had no formal education, 105(65.6%) had more than secondary education, 70(43.8%) were civil servants and 78(48.8%) had no children (Table 1). Also, 49(30.6%) of the respondents were involved in the slaughtering of dogs and sale of the dog meat while 111(69.4%) were involved in handling and consumption of processed dog meat. Ten (20.4%) of the 49 respondents involved in slaughtering reported having been bitten by a dog while 30(27.0%) of the 111 respondents involved in handling and consumption of dog meat reported having been bitten by a dog (Table 2). The reasons for consumption of dog meat as reported by the respondents include: security and spiritual purposes (7.5%), dog meat being special and delicious (13.1%), dog meat being a good source of protein (21.9%) and dog meat being medicinal (9.4%). No specific reasons were given by 20(12.5%) of the respondents.

**Table 1:** Demographic characteristic of respondents in the study areas

	Total number (%) of respondents n=160	Zaria n=100 (%)	Kafanchan n=60 (%)
**Age**			
<19	8(5.0)	3(3.0)	5(8.3)
20-30	65(40.6)	26(26.0)	39(65.0)
31-40	32(20.0)	24(24.0)	8(13.3)
>40	55(34.4)	47(47.0)	8(13.3)
**Gender**			
Male	145(90.6)	91(91.0)	54(90.0)
Female	15(9.4)	9(9.0)	6(10.0)
**Marital status**			
Single	78(48.8)	32(32.0)	46(76.7)
Married	80(50.0)	66(66.0)	14(23.3)
Widowed	2(1.2)	2(2.0)	0(0)
Divorced	0(0)	0(0)	0(0)
**Occupation**			
Unemployed	54(33.8)	20(20.0)	34(56.7)
Civil servant	70(43.8)	59(59.0)	11(18.3)
Businessman/woman	23(14.4)	12(12.0)	11(18.3)
Farmer	13(8.1)	9(9.0)	4(6.7)
**Level of education**			
No formal education	4(2.5)	2(2.0)	2(3.3)
Primary	10(6.2)	9(9.0)	1(1.7)
Secondary	41(25.6)	31(31.0)	10(16.7)
Tertiary	105(65.6)	58(58.0)	47(78.3)
**Number of children**			
None	78(48.8)	33(33.0)	45(75.0)
1	14(8.8)	12(12.0)	2(3.3)
2	10(6.2)	7(7.0)	3(5.0)
3	16(10.0)	11(11.0)	5(8.3)
>3	42(26.2)	37(37.0)	5(8.3)
**Religion**			
Christian	157(98.1)	97(97.0)	60(100)
Islam	2(1.2)	2(2.0)	0(0)
Others	1(0.6)	1(1.0)	0(0)

**Table 2:** Dog ownership status and history of dog bite among processors and consumers of dog meat in Zaria and Kafanchan, Kaduna State, Nigeria

	Processing and Consumption n=49	Consumption only n=111	Chi-square *p*-value	Crude OR (95% CI on OR)
**Number of dogs owned**			0.360	
None	20(27.4%)	53(72.6%)		
1	13(34.2%)	25(65.8%)		
2	7(23.3%)	23(76.7%)		
3	6(42.9%)	8(57.1%)		
>3	3(60.0%)	2(40.0%)		
**Like/keeping dogs**			0.260	0.590(0.234-1.488)
Yes	40(29.0%)	98(71.0%)		
No	9(40.9%)	13(59.1%)		
**Bitten by dog before**			0.373	0.692(0.308-1.558)
Yes	10(25.0%)	30(75.0%)		
No	39(32.5%)	81(67.5%)		

OR- Odds ratio; 95% CI- 95% Confidence interval.

### 3.2 Knowledge of Rabies

The mean knowledge score of the respondents was 9.71 out of the 18 items scored. Forty six (21.8%) of the respondents indicated that rabies cannot kill, 89(55.6%) that rabies can affect all animals and 123(76.9%) knew that dogs are the common source of rabies in Nigeria (Table 3). Only 105(65.6%) of the respondents knew that rabies can affect humans; 60(37.5%) agreed that dog meat processors were at risk of getting rabies and 63.8% reported that a rabid dog could be consumed without any problems. In addition, 110(68.8%) did not have adequate knowledge of the clinical signs of rabies in dogs. However, 73(45.6%) knew it was against the law not to vaccinate dogs (Table 3)

**Table 3:** Assessment of knowledge of the respondents in Zaria and Kafanchan on rabies

Items	Number of respondents (%)
Rabies cannot kill	
Yes	46 (28.8)
No/Don’t know	114 (71.2)
Rabies infects all animals	
Yes	89 (55.6)
No/Don’t know	71 (44.4)
Dogs are common source of rabies in Nigeria	
Yes	123 (76.9)
No/Don’t know	37 (23.1)
Rabies infects all Humans	
Yes	105 (65.6)
No/Don’t know	55 (34.4)
Slaughterers and processors of dog meat are at risk of rabies	
Yes	60 (37.5)
No/Don’t know	100 (62.5)
Rabid animal can be consumed	
Yes	102 (63.8)
No/Don’t know	58 (36.2)
Sudden aggression by an initially friendly dog may be rabies	
Yes	110 (68.8)
No/Don’t know	50 (31.2)
It is against the law not to vaccinate your dog	
Yes	73 (45.6)
No/Don’t know	87 (54.4)
Dog registration and licensing cannot control rabies	
Yes	60 (37.5)
No/Don’t know	100 (62.5)

Compared to Zaria, the frequency of respondents with adequate knowledge was higher in Kafancha; however, the difference was not statistically significant (p>0.05). The proportion of farmers with adequate knowledge was higher than any other occupation group (Table 4). The proportions of respondents with adequate knowledge increased with the level of education with respondents who had attended tertiary level schools having the highest frequency (59.0%).

**Table 4:** Assessment of categorised knowledge scores based on demographic variables of respondent from the study areas

Demographic Variables	Knowledge score remark (Category)		
	Unacceptable (0-9)	Acceptable (10-18)	Crude OR (95% CI on OR)
**Location**			
Zaria	48(48.0%)	52(52.0%)	1.388(0.724-2.649)
Kafanchan	24(40.0%)	36(60.0%)
**Age**			
<19	2(25.0%)	6(75.0%)
20-30	30(46.2%)	35(53.8%)	0.389(0.073-2.649)
31-40	16(50.0%)	16(50.0%)	0.333(0.058-1.907)
>40	24(43.6%)	31(56.4%)	0.431 (0.080-2.326)
**Gender**			
Male	66(45.5%)	79(54.5%)	1.253(0.424-3.703)
Female	6(40.0%)	9(60.0%)
**Marital status**			
Single	35(44.9%)	43(55.1%)	
Married	36(45.0%)	44(55.0%)	0.995 (0.531-1.862)
Widowed	1(50.0%)	1(50.0%)	0.814 (0.049-13.490)
**Occupation**			
Unemployed	22(40.7%)	32(59.3%)	
Civil servant	37(52.9%)	33(47.1%)	0.613 (0.299-1.257)
Businessman/woman	10(43.5%)	13(56.5%)	0.894 (0.333-2.398)
Farmer	3(23.1%)	10(76.9%)	2.292 (0.565-9.291)
**Level of education**			
No formal education	3(75.0%)	1(25.0%)	
Primary	6(60.0%)	4(40.0%)	2.000 (0.150-26.740)
Secondary	20(48.8%)	21(51.2%)	3.150 (0.302-32.850)
Tertiary	43(41.0%)	62(59.0%)	4.326 (0.435-42.990)
**Number of children**			
None	35(44.9%)	43(55.1%)	
1	9(64.3%)	5(35.7%)	0.452 (0.139-1.473)
2	5(50.0%)	5(50.0%)	0.814 (0.218-3.039)
3	5(31.2%)	11(68.8%)	1.791 (0.568-5.641)
>3	18(42.9%)	24(57.1%)	1.085 (0.509-2.313)

### 3.3 Attitudes towards Rabies

The mean attitude score was 6.38 out of 9 items indicating that the respondents had moderately positive attitude towards the disease. Respondents from Kafanchan were 3 times more likely to have positive attitude than those from Zaria (OR=3.00, 95% CI on OR 0.96, 9.33). Older respondents were more likely to develop positive attitudes than those aged less than 19 years. Females and the unemployed also had positive attitudes. Having positive attitudes increased with the level of education and those with tertiary education were 32 times more likely to have positive attitudes than those no formal education (Table 5).

**Table 5:** Assessment of categorized attitude scores based on demographic variables of respondents from study areas

Demographic Variables	Attitude score remark (Category)		
	Unacceptable (0-4)	Acceptable (5-9)	Crude OR (95%CI on OR)
**Location**			
Zaria	18(18.0%)	82(82.0%)	3.073 (0.987-9.566)
Kafanchan	4(6.7%)	56(93.3%)	
**Age**			
<19	2(25.0%)	6(75.0%)	
20-30	5(7.7%)	60(92.3%)	4.000 (0.634-25.240)
31-40	4(12.5%)	28(87.5%)	2.333 (0.345-15.800)
>40	11(20.0%)	44(80.0%)	1.333 (0.236-7.531)
**Gender**			
Male	21(14.5%)	124(85.5%)	2.371 (0.296-18.990)
Female	1(6.7%)	14(93.3%)	
**Marital status**			
Single	7(9.0%)	71(91.0%)	
Married	15(18.8%)	65(81.2%)	0.427 (0.164-1.114)
Widowed	0(0%)	2(100.0%)	2.097 (0.004-1250.000)
**Occupation**			
Unemployed	2(3.7%)	52(96.3%)	
Civil servant	13(18.6%)	57(81.4%)	0.169 (0.036-0.783)*
Businessman/woman	5(21.7%)	18(78.3%)	0.138 (0.025-0.777)*
Farmer	2(15.4%)	11(84.6%)	0.212 (0.027-1.668)
**Level of education**			
No formal education	3(75.0%)	1(25.0%)	
Primary	4(40.0%)	6(60.0%)	4.500 (0.337-60.150)
Secondary	6(14.6%)	35(85.4%)	17.500 (1.551-197.400)
Tertiary	9(8.6%)	96(91.4%)	32.000 (3.009-340.300)
**Number of children**			
None	5(6.4%)	73(93.6%)	
1	6(42.9%)	8(57.1%)	0.091 (0.023-0.368)*
2	1(10.0%)	9(90.0%)	0.616 (0.065-5.884)
≥ 3	1(2.04%)	48(97.96%)	3.288 (0.372 – 29.020)

* significant at p<0.05

### 3.4 Practices towards Rabies

Most respondents (>90%) had acceptable practice scores with mean of 8.34 out of 12 items. Having acceptable practice score decreased with age to some extent but increased with the level of education. However, the associations of age and of level of education with categorized practice scores were not statistically significant (Table 6). About 83.1% the respondents indicated that dog handlers should wear protective clothing, 65.0% indicated that dog bite wounds should be washed with soap and water and 65.0% reported that they would treat dog bite victims with traditional herbs (Table 7). About 96.9% of the respondents agreed that it was good to vaccinate dogs against rabies (Table 7).

**Table 6:** Assessment of categorized practice scores based on demographic variables of respondents from study area

Demographic variables	Practice score remark (Category)		
	Unacceptable (0-5)	Acceptable (6-12)	Crude OR (95% CI on OR)
**Location**			
Zaria	5(5.0%)	95(95.0%)	1.000 (0.230-4.343)
Kafanchan	3(5.0%)	57(95.0%)	
**Age**			
<19	1(12.5%)	7(87.5%)	
20-30	2(3.1%)	63(96.9%)	4.500 (0.361-56.170)
31-40	1(3.1%)	31(96.9%)	4.429 (0.246-79.740)
>40	4(7.3%)	51(92.3%)	1.821 (0.177-18.710)
**Gender**			
Male	7(4.8%)	138(95.2%)	0.710 (0.081-6.196)
Female	1(6.7%)	14(93.3%)	
**Marital status**			
Single	4(5.1%)	74(94.9%)	
Married	4(5.0%)	76(95.0%)	1.027 (0.245-4.259)
Widowed	0(0.0%)	2(100.0%)	1.162 (0.002-714.400)
**Occupation**			
Unemployed	0(0.0%)	54(100.0%)	
Civil servant	2(2.9%)	68(97.1%)	
Businessman/woman	5(21.7%)	18(78.3%)	0.106 (0.019-0.591)*
Farmer	1(7.7%)	12(92.3%)	0.353 (0.030-4.205)
**Level of education**			
No formal education	1(25.0%)	3(75.0%)	
Primary	1(10.0%)	9(90.0%)	3.000 (0.140-64.270)
Secondary	3(7.3%)	38(92.7%)	4.222 (0.330-54.090)
Tertiary	3(2.9%)	102(97.1%)	11.330 (0.896-143.40)
**Number of children**			
None	1(1.3%)	77(98.7%)	
1	2(14.3%)	12(85.7%)	0.078 (0.007-0.927)*
2	1(10.0%)	9(90.0%)	0.117 (0.007-2.034)
3	2(12.5%)	14(87.5%)	0.091 (0.008-1.072)
>3	2(4.8%)	40(95.2%)	0.026 (0.023-2.952)

* significant at p<0.05

**Table 7:** Assessment of practice of the respondents in study areas towards rabies

	Number of respondents (%)
Dog handlers should wear protective clothing	
Yes	133 (83.1)
No/Undecided	27 (16.9)
Dog handlers should take human anti-rabies vaccine	
Yes	136 (85.0)
No/Undecided	24 (15.0)
Wash dog bite wounds with soap and water	
Yes	104 (65.0)
No/Undecided	56 (35.0)
It is good to vaccinate your dog(s)	
Yes	155 (96.9)
No/Undecided	5 (3.1)
It is good to have a cage for your dog(s)	
Yes	146 (91.2)
No/Undecided	14 (8.8)
It is not a good practice to castrate your dog(s)	
Yes	56 (35.0)
No/Undecided	104 (65.0)
Do nothing to a dog bite victim	
Yes	16 (10.0)
No	144 (90.0)
Take a dog bite victim to a medicine/patent store	
Yes	113 (70.6)
No	47 (29.4)
Use traditional herbs to treat dog bite victim	
Yes	104 (65.0)
No	56 (35.0)
Take dog bite victim to the Veterinary clinic	
Yes	129 (80.6)
No	31 (19.4)
Take dog bite victim to the hospital	
Yes	128 (80.0)
No	32 (20.0)

### 3.5 Relationship between Attitude and Practice and Knowledge

Results of multiple regression analysis indicated that there are significant correlations between knowledge and attitude scores (r=0.49) and knowledge and practice scores (r=0.43) at p<0.001. This indicates that respondents with higher knowledge scores had more positive attitude and more acceptable practices towards rabies prevention (Table 8).

**Table 8:** Multiple regression analysis summaries for attitude and practice scores predicting knowledge score (n=160)

Variable	Unstandardized coefficient, B.	Standard error of B, SEB.	Standardized coefficient, β.
Attitude score	0.49	0.15	0.25*
Practice score	0.43	0.17	0.20*
Constant	3.06	1.41	

Note: F= (2,157) =12.90, P=0.000

* P<0.05. F= value from ANOVA

### 3.6 Detection of Rabies Antigen

Of the 154 brain samples examined, 6(3.9%) were positive for rabies antigen. Four (5.4%) of the 74 samples from Zaria and two (2.5%) of the 80 samples from Kafanchan were positive (Table 9).

**Table 9:** Percentage of sample collected from slaughtered dogs in study area that are positive for rabies virus antigen

Location	Number of samples collected	Number of collected samples positive	Percentage (%)
Zaria Kafanchan	74 80	4 2	5.4 2.5
Total	154	6	3.9

## 4. Discussion

A high proportion of the respondents were males because males are the ones involved in slaughtering of dogs and they are the ones who frequent drinking parlours where dog meat is sold. A good number of them were civil servants who like to relax with dog meat after work. About 71.2% of the study population responded no or don’t know when asked if rabies can kill, while 55.6% knew all animals can be infected with rabies. It is likely that they believe rabies can be cured which is not the case and they need to be properly educated to ensure they take potential exposures seriously. Even though handling raw dog meat is risky, the risk is higher in those who are involved in the actual slaughtering which in this study formed about 30% of the respondents. In some cases bites occur when the dogs are being restrained for slaughter. It is interesting to note some people eat dog meat as a remedy for a various ailments. Even though about 76.9% of the respondents knew the dogs are the common source of rabies in Nigeria, only 37.5% agreed that dog meat processors were at risk of getting rabies. That means that over 60% respondents did not know the risk involved in dog meat processing and is probably why they take no safety precautions when handling raw dog meat. Many people associate rabies with dogs and even the native names for rabies in the local vernacular is suggestive of the role of dogs in the disease. Some of these names are *ciwon haukan kare*, *ara nnkita* and *dibolougi* in the three major Nigerian languages namely Hausa, Igbo and Yoruba respectively, which translate to mad dog disease. The information gap here may be that they do not know other means of transmission apart from dog bite and so they do not link other possible exposures with the disease transmission.

Zaria has more veterinary infrastructure than Kafanchan yet the proportion of respondents with adequate knowledge about rabies was higher in Kafanchan probably because the dog population is higher in Kafanchan and there are generally more rabies cases in dogs than in Zaria (Ezeokoli & Umoh, 1987). Farmers had higher level of awareness than other occupational groups because they work in rural areas where rabies cases are more rampant making them to be familiar with the disease.

The results also indicated that the more educated the person is, the more knowledgeable he is about rabies. This is because educated persons have access to print and electronic media which occasionally give information on rabies. Our study found that, in general, dog meat processors and consumers had positive attitudes towards handling exposure situations. Respondents from Kafanchan had more positive attitudes than those from Zaria probably because they were more aware of rabies due to frequent occurrence of cases in the area (Ezeokoli & Umoh, 1987). Individuals who were more aware tended to develop more positive attitude as shown by older respondents and by more educated respondents. Most of the respondents seem to know what to do to prevent rabies and educated respondents had more acceptable practice scores. Even though a significant proportion of the respondents (83.1%) advised that dog handlers and processors of dog meat should wear protective clothing, none of them was observed to wear any protective clothing during slaughtering and processing of dog meat. There is great need to press upon them to protect themselves during slaughter.

Even though the rate of detection of rabies antigen in the brains of the slaughtered dogs was lower than the rates observed by Garba et al. (2007), Akombo (2009) etc, the risk of exposure of the dog meat processors to rabies still exists. This is especially so since the slaughtering is a continuous process and the risk of exposure to rabies increases with the frequency of being involved in dog meat processing

In general, the results revealed that the dog meat processors and consumers have deficiencies in respect to their knowledge of rabies. These tend to result in negative attitudes and unsafe practices during dog meat processing; thus increasing the risk of exposure to rabies since some of the slaughtered dogs may be infected with rabies virus.
